# Evaluation of the Cytotoxicity of Ayahuasca Beverages

**DOI:** 10.3390/molecules25235594

**Published:** 2020-11-28

**Authors:** Ana Y. Simão, Joana Gonçalves, Ana Gradillas, Antonia García, José Restolho, Nicolás Fernández, Jesus M. Rodilla, Mário Barroso, Ana Paula Duarte, Ana C. Cristóvão, Eugenia Gallardo

**Affiliations:** 1 Centro de Investigação em Ciências da Saúde (CICS-UBI), Universidade da Beira Interior, Avenida Infante D. Henrique, 6200-506 Covilhã, Portugal; ana.simao@ubi.pt (A.Y.S.); janitagoncalves@hotmail.com (J.G.); jose.restolho@ubi.pt (J.R.); apcd@ubi.pt (A.P.D.); 2 Laboratório de Fármaco-Toxicologia, UBIMedical, Universidade da Beira Interior, Estrada Municipal 506, 6200-284 Covilhã, Portugal; 3 CEMBIO, Center for Metabolomics and Bioanalysis, Facultad de Farmacia, Universidad San Pablo CEU, CEU Universities, Campus Monteprincipe, Boadilla del Monte, 28668 Madrid, Spain; gradini@ceu.es (A.G.); antogar@ceu.es (A.G.); 4 Cátedra de Toxicología y Química Legal, Laboratorio de Asesoramiento Toxicológico Analítico (CENATOXA), Facultad de Farmacia y Bioquímica, Universidad de Buenos Aires, Junín 956, Ciudad Autónoma de Buenos Aires (CABA), Buenos Aires C1113AAD, Argentina; nfernandez@ffyb.uba.ar; 5 Materiais Fibrosos e Tecnologias Ambientais—FibEnTech, Departamento de Química, Universidade da Beira Interior, Rua Marquês D’Ávila e Bolama, 6201-001 Covilhã, Portugal; rodilla@ubi.pt; 6 Instituto Nacional de Medicina Legal e Ciências Forenses, Serviço de Química e Toxicologia Forenses, Delegação do Sul, Rua Manuel Bento de Sousa n.°3, 1169-201 Lisboa, Portugal; mario.j.barroso@inmlcf.mj.pt; 7 NEUROSOV, UBIMedical, Universidade da Beira Interior, Estrada Municipal 506, 6200-284 Covilhã, Portugal

**Keywords:** ayahuasca, cytotoxicity, mesencephalic dopaminergic neurons, *N*,*N*-dimethyltryptamine, beta-carboline alkaloids

## Abstract

Ayahuasca is a beverage consumed at shamanic ceremonies and currently has gained popularity on recreational scenarios. It contains beta-carboline alkaloids and *N*,*N*-dimethyltryptamine, which possesses hallucinogenic effects. Only a few studies have elicited the psychoactive effects and the dose of such compounds on neurological dopaminergic cells or animals. In this work, we aimed to study the cytotoxic effects of these compounds present in ayahuasca beverages and on five different teas (*Banisteriopsis caapi*, *Psychotria viridis*, *Peganum harmala*, *Mimosa tenuiflora* and Dc Ab (commercial name)) preparations on dopaminergic immortalized cell lines. Moreover, a characterization of the derivative alkaloids was also performed. All the extracts were characterized by chromatographic systems and the effect of those compounds in cell viability and total protein levels were analyzed in N27 dopaminergic neurons cell line. This is the first article where cytotoxicity of ayahuasca tea is studied on neurological dopaminergic cells. Overall, results showed that both cell viability and protein contents decreased when cells were exposed to the individual compounds, as well as to the teas and to the two mixtures based on the traditional ayahuasca beverages.

## 1. Introduction

Ayahuasca is a psychoactive beverage prepared traditionally from a brew mixture of the leaves and stems of *Psychotria viridis* and *Banisteriopsis caapi*, respectively, being originally consumed by indigenous Amazonian tribes for ritual and medicinal purposes [1,2,3,4]. Over the years, its use has spread to other groups not only as a source of personal growth and spiritual connection, but also in recreational scenarios, hence becoming a serious concern for public heatlh due to its toxicity. In addition, the hypothesis of its use as a potencial therapeutic drug is being discussed [4,5,6,7]. The main compounds of this tea-like preparation are *N*,*N*-dimethyltryptamine (DMT) and beta-carboline alkaloids or harmala alkaloids, such as harmine (HMN), tetrahydroharmine (THH) and harmaline (HML) [8,9,10,11]. Due to the fact that these compounds are present in several different plants, it is possible to replace those used in the traditional preparation for other plants, such as *P. harmala* and *Mimosa tenuiflora* also known as *M. hostilis*, amongst others [3,12,13,14,15]. Beta-carboline alkaloids are monoamine-oxidase (MAO) inhibitors, thus allowing DMT, which is the main psychoactive compound in the ayahuasca beverages, to exert its psychoactive and hallucinogenic effects on the central nervous system (CNS) [11,16]. Since DMT presents a similar structure to serotonin [17], its main biochemical effects are on the serotoninergic receptors, such as 5HT_2_A, which are known to be the site of action of hallucinogenic drugs [18]. In addition, the consumption of ayahuasca beverages with high contents of DMT, increases the levels of serotoninergic receptors, but also the intake of MAO inhibitors leading to accumulation of dopamine, which is associated to the sensations of pleasure and reward, but also addiction [18]. Nevertheless, the accumulation of dopamine may contribute to its auto-oxidation, which in turn it is well known to account for the formation of neuromelanin and consequent neurodegeneration of dopaminergic neurons [19,20]. Although consumers defend the use, its effects on the CNS are not fully understood yet. Currently, there are few studies on the toxicological effects of ayahuasca [11] and to date no study has evaluated the cytotoxic effects of ayahuasca teas on dopaminergic neuronal cells; also, the majority of investigations performed so far are based on clinical trials or even on surveys. In the present study, the aim was to evaluate the toxic effects on dopaminergic rat cells of HMN, HML, THH, DMT and five different plants, which are used to prepare ayahuasca beverages, and, the tradional mixtures of *P. viridis*/*B. caapi* and *P. viridis*/*P. harmala*. To the best of our knowledge, this is the first study to investigate cytotoxicity of ayahuasca compounds and commercial teas on dopaminergic neuronal cells.

## 2. Results and Discussion

### 2.1. Characterization of Alkaloid Derivatives and Other Compounds

All five plants (*P. viridis*, *B. caapi*, *P. harmala*, *M. tenuiflora* and Dc Ab (commercial name)) were characterized in terms of alkaloid derivatives present in them.

**Figure 1:**
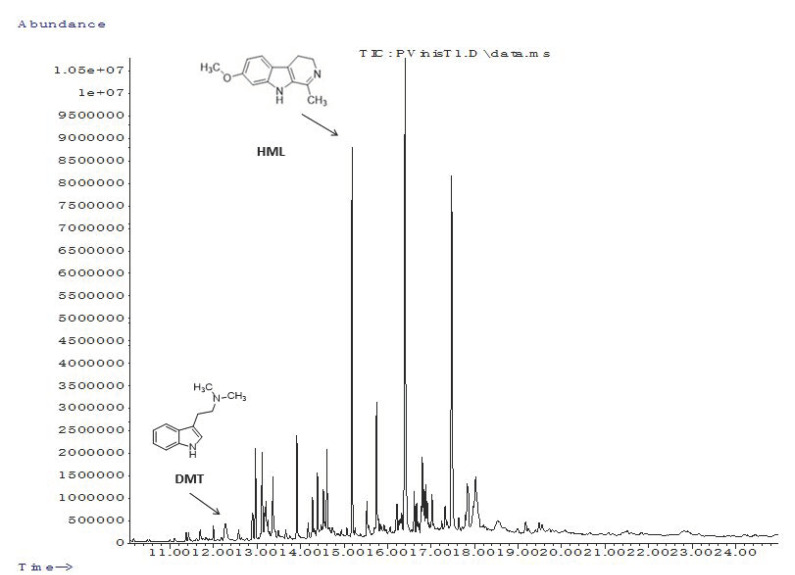
Chromatogram of *Psychotria viridis*.

Through GC–MS analysis in scan (Figure 1) and SIM modes it was possible to identify and determine the concentrations of DMT, HMN, THH and HML. The concentrations found were 2.06 µg/mL of DMT and 0.20 µg/mL of HML for *P. viridis*. In the case of *M. tenuiflora*, the concentration of DMT was inferior (1.70 µg/mL), and also 0.74 µg/mL of HMN was found. As for *B. caapi*, *P. harmala* and Dc Ab, all plant extracts contained THH, HML and HMN on different concentration levels. The first, contained 1.22, 0.99 and 12.81 µg/mL, respectively. As for *P. harmala*, the levels were as follows, 7.46, 26.95 and 24.27 µg/mL, it is important to note that this plant extract was the one that presented the highest levels of beta-carnoline alkaloids. Finally, Dc Ab contained 1.11, 0.30 and 0.20 µg/mL of THH, HML and HMN, respectively [21].

However, due to the characteristics and limitations of GC, a more complete characterization was carried out through UHPLC/QTOF-MS through a non-targeted metabolomic approach mainly focused on secondary metabolites such as alkaloids and phenolic compounds.

**Figure 2:**
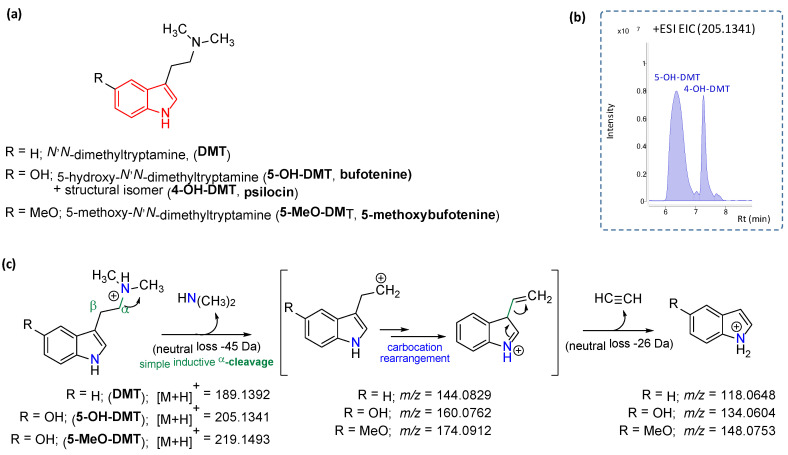
(**a**) Structures of the tryptamine alkaloids detected from the methanolic extracts. (**b**) Extracted ion chromatograms (EICs) at *m*/*z* 205.1341. A complete chromatographic separation was observed for bufotenine (5-HO-DMT) and its structural isomer, psilocin (4-HO-DMT). (**c**) Detail of the main fragment ions and the fragmentation pathway proposed.

**Figure 3:**
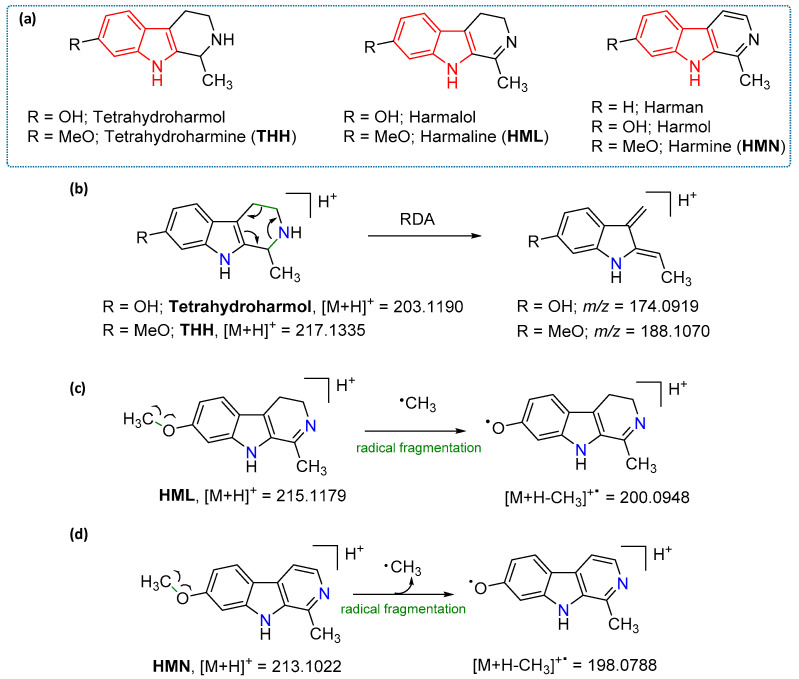
(**a**) Structures of the beta-carboline alkaloids detected from the methanolic extracts. (**b**–**d**). Detail of the main fragment ions and the fragmentation pathways proposed.

The major phenolic compounds were identified by comparison with authentic reference standards (Table 1). Putative identification of the alkaloids detected (Table 1 and Figure 2 and Figure 3) was carried out by comparing their retention times, accurate masses (±5 ppm error) against online databases, such as FooDB (http://foodb.ca), CEU massmediator (http://ceumass.eps.uspceu.es/mediator) and scientific bibliography. The in-source fragmentation gives a pattern of fragments based on the dissociation of the protonated pseudomolecular ions [M + H]^+^ when a 175 V fragmentor voltage is applied. The characteristic fragment ions were key to study and understand the fragmentation mechanisms and to support metabolite annotation with a higher confidence level (Figure 2 and Figure 3). Besides they are in agreement with previous studies [22,23,24].

Results show that *P. viridis*, *M. hostilis* and Dc Ab are the ones that contain the highest levels of tryptamine alkaloids, mainly DMT and 5-OH-DMT [25]. On the other hand, *B. caapi* and *P. harmala* contain the highest levels of beta-carboline harmala alkaloids, mainly HML and HMN (Figure 3), as it was expected [25].

The main fragmentation pathway proposed for tetrahydroharmol and THH involves a retro-Diels-Alder (RDA) mechanism to the tetrahydro ring, Figure 3 [23,24]. A characteristic methyl radical loss is also observed for THH, HML and HMN, Figure 3.

Table 1 includes also relevant information about phenolic compounds. The profiles of these compounds in each plant variety of ayahuasca beverages was recently published by our group, where, in addition to determining the concentration of phenolic compounds, the antioxidant activity, anti-inflammatory activity and antimicrobial properties were also evaluated [26].

### 2.2. Cytotoxicity Evaluation

**Figure 4:**
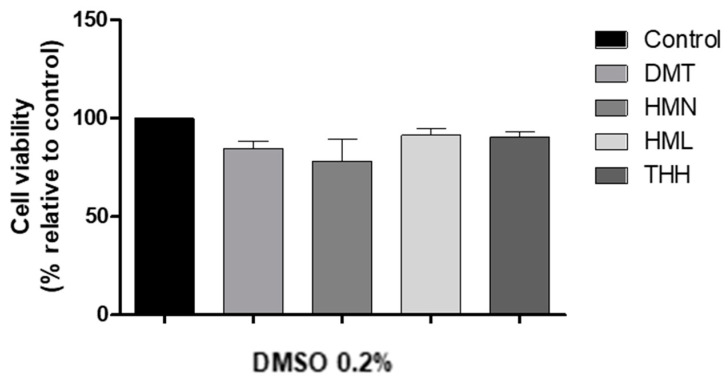
The effect of 0.2% of DMSO on N27 cell viability (24 h). The highest concentration used as a vehicle in the evaluation of the respective compounds was 0.2% of DMSO (Control—untreated cells (100 ± 0.00), DMT (1 µM; 84.86 ± 6.29), HMN (10 µM; 78.04 ± 20.13), HML (80 µM; 91.40 ± 5.89) and THH (10 µM; 90.49 ± 4.36); *n* = 3, values are shown as mean ± SEM; no significant difference was observed when performing ANOVA followed by Dunnett’s multiple comparison test).

Firstly, a cytotoxic evaluation of the individual compounds (DMT, HMN, HML and THH) was performed. Since they are water insoluble, DMSO was used as a vehicle. We used 0.2% of DMSO in order to guarantee the solubility of the standard compounds. Therefore, a preliminary study was carried out in order to assess if the solvent could induce cytotoxicity on its own (Figure 4). It is known that high amounts of this solvent (≥ 1–5%) can induce cell death [27]. It was tested the toxic effect on N27 cells of the highest percentage of DMSO, i.e., 0.2%, that was used as a vehicle. As observed on Figure 4, despite existing a decrease in cell viability, when compared to untreated cells (control), no significant difference is observed. Therefore, this suggest that 0.2% of DMSO did not induce a reduction of cell viability, ruling out its contribution to the putative toxic effect of the compounds under study. For this reason, it could be assumed that, if a compound or even the teas induce an increase of cell death, this may be caused by the compound itself and not due to the presence of the vehicle.

**Figure 5:**
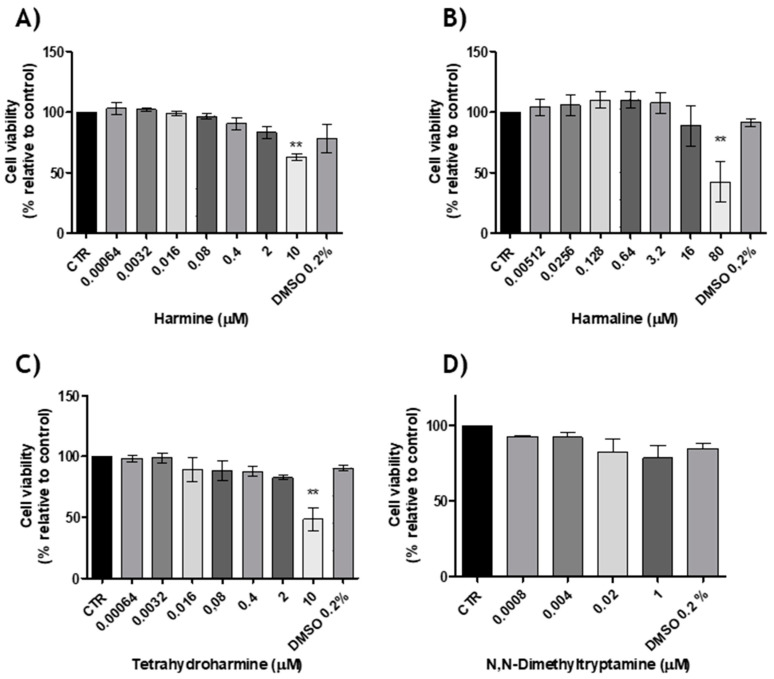
The effects of (**A**) HMN, (**B**) HML, (**C**) THH and (**D**) DMT on N27 cell line viability (24 h), (*n* = 3, values are shown as mean ± SEM ** indicates values that are significantly different from control *p* < 0.01, one-way analysis of variance followed by Bonferroni’s multiple comparison test). CTR: Control.

After discarding the possibility of vehicle-induced toxicity, the cytotoxic evaluation was performed for all the individual compounds, that is, HMN, HML, THH and DMT at different concentrations. Results are shown in Figure 5.

Regarding the response induced by HMN on N27 cells, a range of concentrations from 0.00064 to 10 µM was assessed. It was observed that from 0.00064 to 2 µM, cell viability was not affected during the time of cell incubation (24 h) and no statistical differences were noticed. Nonetheless, for cells incubated with 10 µM, we were able to see a decrease in cell viability to 62.85% ± 4.78% when compared to the control (0 µM) culture (Figure 5A). The same concentrations were studied for THH and the results were similar, that is, when cells were incubated with 10 µM of this compound, a decrease in cell viability was observed (48.25% ± 16.21%) in comparison to the control. For all other concentrations (0.00064–2 µM), cell viability was not affected (Figure 5C). As for HML, a range of concentration levels from 0.00512 to 80 µM was evaluated. Once again, only at the highest concentration level, cell viability was affected, namely when cells were exposed to 80 µM of HML (Figure 5B). As a whole, the results suggest that a higher concentration of these compounds may induce dopaminergic neuronal toxicity. Finally, for DMT the concentration levels studied covered were from 0.0008 to 1 µM. In this case, for all concentrations no statistical differences were observed when cells were exposed to this compound (Figure 5D). These results are similar according to the literature, once the psychotropic effects of ayahuasca are only achieved when there is an interaction between DMT and beta-carbolines [11], on the other hand, recent studies showed that beta-carbolines have neuroprotective effects [28].

**Figure 6:**
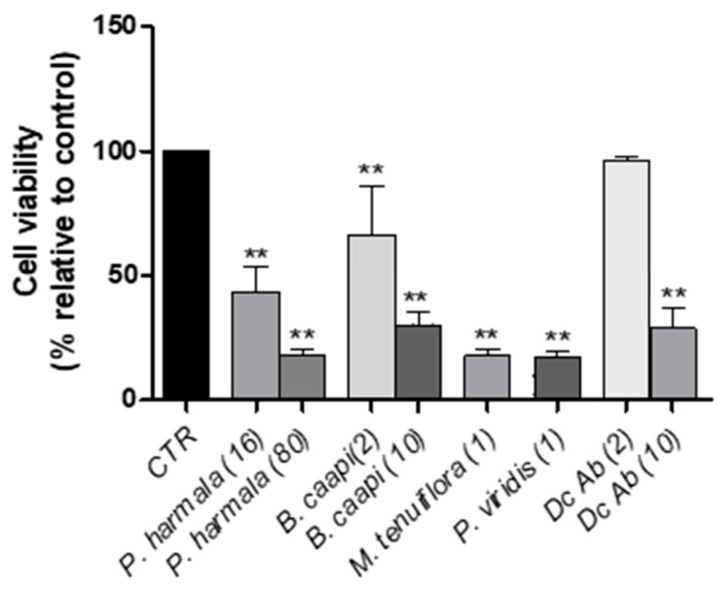
The effects of *P. harmala* (16 and 80 µM), *B. caapi* (2 and 10 µM), *M. tenuiflora* (1 µM), *P. viridis* (1 µM) and Dc Ab (2 and 10 µM) on N27 cell viability (24 h); CTR—control, (*n* = 3, values are shown as mean ± SEM ** indicates values that are significantly different from control *p* < 0.01, one-way analysis of variance followed by Dunnett’s multiple comparison test).

Once obtaining the results for the individual chemical compounds present in tea, the dopaminergic neuronal cytotoxic effect of tea extracts was analyzed. The concentrations were chosen based on the results of the cytotoxic evaluation of each compound (the highest concentration causing less cell viability and the previous). A total of five tea plants (*P. viridis, B. caapi, P. harmala, M. tenuiflora* (also known as *M. hostilis*) and Dc Ab (commercial name)) and two different mixtures of plants were incubated with N27 cells to evaluate their effects on cell viability (Figure 4). Each plant extract quantity was chosen according to the concentrations of the standards that caused less cell viability (Figure 5) and the concentration of compounds present in each of them after GC–MS analysis. The cytotoxic evaluation of the tea extracts is present on Figure 6.

The evaluation of *P. harmala*’s effect on N27 cells, at two different concentrations (16 and 80 µM of HML), showed a significant decrease in cell viability, suggesting a neurotoxic effect. In fact, at the highest concentration studied, the observed cell viability was of 17.72% ± 2.29% when compared to the control culture. This cytotoxic effect, when compared with the effects observed with the individual compounds, may result from the interaction between the beta-carbolines present in this tea (THH, HMN and HML), exerting synergetic and negative effects for these cells.

The same happened for *B. caapi*, i.e., when cells were exposed to 10 µM of HMN (highest concentration) a significant decrease in cell viability was noted. From the quantification results obtained by Simão et al., [21], this tea presents high content in HMN (12.80 µg/mL), still it also contains low traces of THH (1.12 µg/mL) and HML (0.99 µg/mL). Once again, these results suggest that the possible interaction between these compounds may cause the cytotoxic observed effects on N27 cells.

In the case of *M. tenuiflora*, it is known that DMT is part of its [3,14], still on our previous study we were able to quantify 0.74 µg/mL of HMN in this tea [21]. This may suggest the obtained result of incubation cells with this tea at 1 µM of DMT. The same was observed for *P. viridis*, only this time apart from DMT, the tea was also composed of HML [21].

Finally, for Dc Ab, only when cells were exposed to 10 µM of THH, a significant decrease of N27 cell viability was observed.

**Figure 7:**
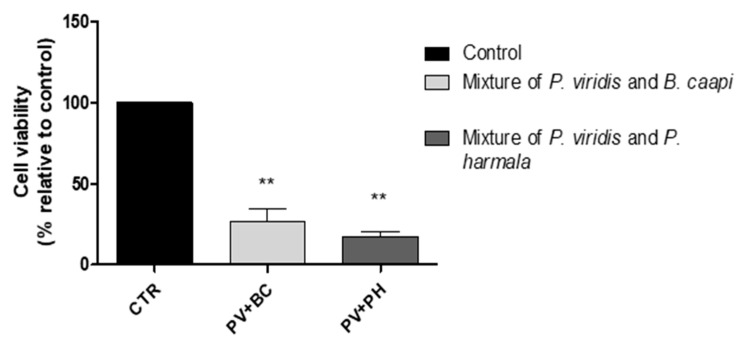
The effects of tea plant extracts mixtures (*P. viridis* plus *B. caapi* and *P. viridis* plus *P. harmala*) on N27 cell viability (*n* = 3). The presented values are presented as mean ± SEM. ** indicates values that are significantly different from control (*p* < 0.05, one-way analysis of variance (ANOVA)). CTR—control.

In addition, when exposing N27 cells were exposed to tea mixtures, a clear decrease in cell viability occurred (Figure 7).

### 2.3. Total Protein Quantification

**Figure 8:**
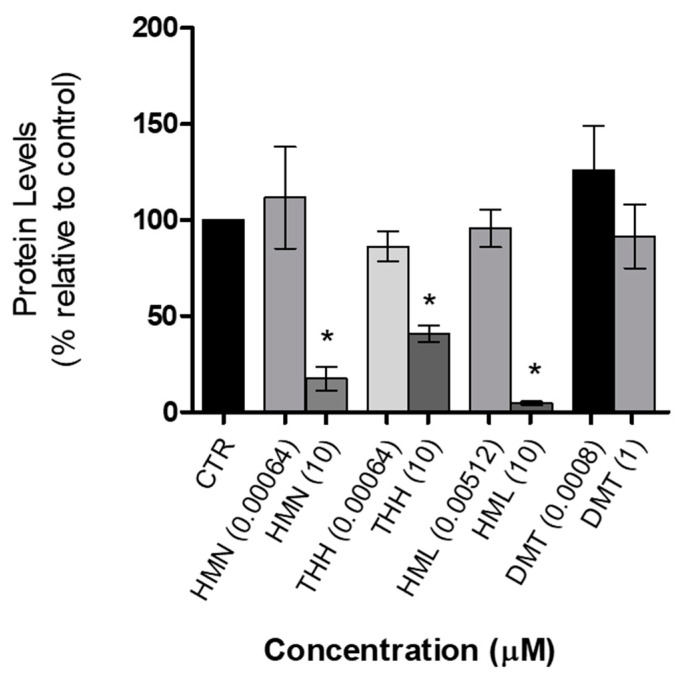
Protein levels when N27 cells are exposed during 24 h to HMN, THH, HML and DMT; CTR (control with untreated cells; 0.59 ± 0.08), HMN 0.00064 µM (0.65 ± 0.04), HMN 10 µM (0.19 ± 0.02), THH 0.00064 µM (0.56 ± 0.09), THH 10 µM (0.32 ± 0.05), HML 0.00512 µM (0.59 ± 0.04) and HML 10 µM (0.14 ± 0.0006); DMT 0.0008 µM (0.73 ± 0.02); DMT 1 µM (0.59 ± 0.03). *n* = 3, values are shown as mean ± SEM * indicates values that are significantly different from control *p* < 0.05, one-way analysis of variance followed by Dunnett’s multiple comparison test. CTR—control.

In order to understand the effect of the studied compounds in the cellular protein metabolism, the total protein levels quantification was performed for the highest and the lowest concentration of each compound individually (Figure 8). This assay allows measuring the total amount of intracellular protein levels, to estimate the total biomass contents of the cells that remained adherent (therefore, viable).

These results were in agreement with those obtained from the cytotoxicity assays. In other words, when cell viability decreased, so did the total cellular proteic contents, namely at the highest concentration levels. Although the number of cells decreased, we did not observe the same relation in the reduction of the total protein contents of the surviving cells. We were able to observe a higher decrease in total protein levels, when compared to the decrease in cell viability. This may be a reflection of a reduction of cell function in the presence of HML. The reduction of functionality is often associated with the decrease in transcription and transduction, which is reflected in a lower amount of total protein production [19]. Moreover, it is also known that oxidated dopamine may interfere with the normal protein degradation system, leading to an increase of degradation [29,30].

Cellular protein levels is determined both by the amounts that are synthetized and the rates of degradation, which in the latter case there are different pathways that explain how this may occur: the ubiquitin–proteasome system, autophagy or by degradation mediated by proteolysis in the lysosome [31]. Although this remains to be evaluated in future studies, the results obtained on the reduction of the total protein content in N27 cells exposed to each compound may be explained by the putative inhibitory effect on the protein synthesis machinery or activator effect on the protein degradation systems, exerted by each compound. For instance, on a study by Valente and coworkers [32], it was shown a relationship between the neurotoxic effects caused by two β-keto amphetamines on SH-SY5Y human dopaminergic cells and autophagy dysregulation. The results obtained by these authors suggest that the neurotoxicity of such compounds lead to the production of reactive oxygen species and consequently to an activation of autophagy, both in a time and concentration-dependent manner. Despite being different compounds from those studied in this work, one can speculate that psychoactive compounds may induce autophagy and downregulate protein levels and ultimately induce cellular apoptosis [32]. Nevertheless, future studies need to be performed to understand if the compounds studied herein affect the autophagy flux.

## 3. Materials and Methods

### 3.1. Sample Preparation

Samples of *P. viridis*, *B. caapi*, *M. hostilis* and *P. harmala*, and the commercial mixture Dc Ab were purchased online from the Shayana Shop (https://www.shayanashop.com, Amsterdam, The Netherlands). Five decoctions of each individual plant were prepared, for which 0.210 g of each sample was macerated in a mortar with a few drops of water. The mixture was transferred to a flask and 250 mL of ultrapure water was added. The flasks were boiled at 100 °C for 4 h. Similarly, two decoctions were prepared, in which two vegetal samples were mixed (*P. viridis* and *B. caapi*; *P. viridis* and *P. harmala*; *M. hostilis* and *B. caapi*; *M. hostilis* and *P. harmala*).

On the one hand, the sample preparation method previously described by Simão et al. [21] was carried out to perform the determination of the major metabolites on the five different plants and in the tea mixtures. The method was performed as follows: after solid phase extraction, the obtained extracts were evaporated to dryness under a steam of nitrogen. After this step, the residues were dissolved in 50 μL of methanol, vortex mixed and 2 μL was injected into the GC–MS system in the SCAN mode.

On the other hand, after the decoctions were prepared and let to cool at room temperature and filtered, 50 mL of each sample was collected into falcon tubes and stored at −20 °C for 12 h and the stored at −80 °C for 24 h. Afterwards, samples were freeze-dried for 5 days.

The analysis of UHPLC-QTOF/MS was performed as follows, addition of 300 μL of methanol to the 30 mg of the tea plant lyophilized powder, followed by a 2 min agitation in a vortex. The mixture was then sonicated for 15 min and centrifuged at 10,000× *g* for 5 min at 4 °C. Supernatants were collected and transferred to Chromacol vials (Thermo Fisher Scientific, Madrid, Spain) for LC/MS analysis. The whole procedure was performed in duplicate.

### 3.2. GC–MS and UHPLC-QTOF/MS Analysis

This analysis was based on the study by Simão et al., [21] and was accomplished using a gas chromatographer (GC) model HP7890B coupled to a mass spectrometer (MS) model 5977A from Agilent Technologies. The separation of the analytes was achieved using a 5% phenylmethylsiloxane (HP-5MS) capillary column (30 m × 0.25 mm; 0.25 μm I.D.) from Agilent Technologies. The carrier gas, helium, was set at a constant flow rate of 0.8 mL/min. The injection volume was 2 μL in the splitless mode. The temperatures of the injector and detector were set at 250 and 280 °C, respectively. Ion source temperature was set at 230 °C and quadrupole at 150 °C. The oven temperature started at 90 °C for 3 min increasing at 15 °C/min up to 300 °C, held for 8 min. The total analysis time was 25 min. The mass spectrometer was operated at 70 eV with a filament current of 300 μA in the positive electron ionization mode. Data was acquired in the scan mode (*m*/*z* 40–550) and the selected ion monitoring (SIM) mode using the ChemStation from Agilent Technologies.

Samples were analyzed using a 1290 Infinity series UHPLC system coupled to a 6545 iFunnel QTOF/MS system (Agilent Technologies, Waldbronn, Germany). For compound separation, 2 μL of extract was injected in a reversed-phase column (Zorbax Eclipse XDB-C18 4.6 mm × 50 mm, 1.8 µm, Agilent Technologies) at 40 °C. The flow rate was 0.5 mL/min, and the mobile phase consisted of 0.1% formic acid in ultrapure water (solvent A) and methanol (solvent B). Gradient elution consisted of 2% B (0–6 min), 2–50% B (6–10 min), 50–95% B (11–18 min), 95% B for 2 min (18–20 min), and returned to the initial conditions from 20–21 min. The total analysis time was 25 min. The detector was operated in full scan mode (*m*/*z* 50–1500), at a rate of 1 scan/s. Accurate mass measurement was assured through an automated calibrator delivery system that continuously introduced a reference solution containing masses of *m*/*z* 121.0509 (purine) and *m*/*z* 922.0098 (HP-921) in the positive ESI mode; and of *m*/*z* 112.9856 (TFA) and *m*/*z* 922.009798 (HP-921) in the negative ESI mode. The capillary voltage was ±4000 V for positive and negative ionization modes. The source temperature was 225 °C. The nebulizer and gas flow rates were 35 psig and 11 L/min respectively; the fragmentor voltage was set to 75 V and a radiofrequency voltage in the octopole (OCT RF Vpp) of 750 V was used. Chromatographic analysis was performed using optimized conditions developed by the Center of Metabolomics and Bioanalysis (CEMBIO) [26,33,34].

LC–MS grade solvents were used, and ultrapure water was obtained from a Milli-Q Plus™ System from Millipore (Milford, MA, USA). Formic acid was purchased from Aldrich (St. Louis, MO, USA).

The MassHunter Workstation Software LC/MS Data Acquisition version B.07.00 (Agilent Technologies) was used to control and for data acquisition. The secondary metabolic profile of methanol extracts was analyzed in both ESI negative and positive modes in the range of *m*/*z* 50–1500 Da.

### 3.3. Biological Activities Evaluation

#### 3.3.1. Cell Treatments

The immortalized rat mesencephalic dopaminergic neuron (N27) cell line was maintained in Roswell Park Memorial Institute (RPMI)-1640 culture medium supplemented with 10% fetal bovine serum (FBS), and a mixture of antibiotics (100 U/mL penicillin and 100 µg/mL streptomycin). Subsequently, the cells were incubated at 37 °C with a humidified atmosphere with 5% CO_2_.

When cells were approximately 90–95% confluent, they were trypsinized and seeded in 96-well plates (1 × 10^4^ cells/well) and were left to adhere for 12–16 h. Then, after reaching 80–90% confluence in the well plates, the medium was replaced and cells were incubated with certain amounts of the most abundant compound in each plant material, assuming that toxicity is due mainly to its action. The used concentrations were 16 and 80 µM of HML (*P. harmala*), 2 and 10 µM of HMN (*B. caapi* and Dc Ab) and 1 µM of DMT (*P. viridis* and *M. tenuiflora*) for 24 h, in the appropriate medium. The tea mixtures (*P. viridis/B. caapi* and *P. viridis/P. harmala*) were prepared by weighing plant material, assuring that the concentrations of DMT and HMN were 1 and 10 µM respectively for *P. viridis/B. caapi* and 1 and 80 µM of DMT and HML for *P. viridis/P. harmala*. The DMSO vehicle present in each extract was kept below 0.2% to prevent solvent-induced cytotoxicity. Each working solution was prepared in NaCl 0.9%. Each experiment was repeated at least three times, independently.

#### 3.3.2. Cellular Viability Assay

The cytotoxicity of the chosen samples was determined by measuring the levels of cell viability using the cell counting kit-8 (CCK-8) assay (Tebu-bio, Lisboa, Portugal) after 24 h of incubation. This assay was based on the reduction of a slightly yellow water-soluble tetrazolium salt, 2-(2-methoxy-4-nitrophenyl)-3-(4-nitrophenyl)-5-(2,4-disulfophenyl)-2H-tetrazolium and monosodium salt (WST-8), into an orange colored product (WST-formazan), which is soluble in the culture medium. Moreover, the quantity of formazan dye formed was directly proportional to the number of living cells, thus allowing determining the number of viable cells through absorbance measurements.

The cells were seeded in 96-well plates (1 × 10^4^ cells/well), which after reaching confluence were exposed to the tea samples dissolved in RPMI-1640 culture medium. At the end of the incubation time, the cell culture medium was removed from the wells and replaced by a mixture of 5 µL of CCK-8 solution and 95 µL of culture medium and incubated again at 37 °C, light protected, for 3 h. Afterwards, the absorbances were measured using a microplate spectrophotometer (xMArk™ Microplate Absorbance Spectrophotometer, BIO-RAD) at 450 nm.

#### 3.3.3. Total Protein Extraction and Quantification

N27 cells were maintained following the same criteria as explained on Section 3.3.1. The cells were then seeded in 6-well plates (2 × 10^5^ cells/well). After 24 h incubation with the highest and lowest concentrations of each tea preparation, the cell medium was removed and cells extracts were collected. In order to obtain cell extracts, cells were scraped and collected in PBS from the culture dish. Afterwards, the resulting suspension was centrifuged at 18,000× *g* for 1 min. The resulting pellet was suspended in 20 μL of radioimmunoprecipitation (RIPA) lysis buffer and cells were let to rest on ice for 15 min. Afterwards, cells were centrifuged at 14,000× *g* at 4 °C for 20 min; the new resulting supernatant was collected for total protein quantification using the Pierce ™ bicinchoninic acid (BCA) protein assay kit.

A calibration curve of bovine serum albumin (BSA) was used to determine the total protein amount in each sample. Therefore, from 100 µL of BSA (2 µg/µL), serial dilutions with water were made (the final concentrations were of 1 μg/μL, 0.5 μg/μL, 0.25 μg/μL, 0.125 μg/μL, 0.0625 μg/μL and 0 μg/μL). A solution of RIPA was diluted 11 times in water on a different Eppendorf tube. All protein samples were also diluted 11 times in water, that is, for 5 µL of protein, 50 µL of water was added into the respective tubes. The estimated volume of BCA reagent need was obtained according to the number of wells, so knowing that each well uses a total volume of 200 μL, the ratio used was 49 parts of reagent A (mixture of sodium carbonate, sodium bicarbonate, BCA and sodium tartrate in 0.1 M of sodium hydroxide) and 1 part of the B reagent (4% of cupric sulphate).

In a 96-plate, for the standard curve 25 μL of RIPA + 25 μL of BSA standards was added to each well. As for our samples, 25 μL of water + 25 μL of each diluted sample was added to each well. All standards and samples were analyzed in duplicate. Following this, 200 μL of the mix (reagent A + B) was added to each well. Then, the plate was incubated for 30 min at 37 °C and kept in the dark. Subsequently, the absorbance was read at 570 nm.

### 3.4. Statistical Analysis

Experiments were performed in three sets of independent experiments (*n*), and four replicates. The results are presented in percentage of controls, according to the following expression:

% CTR = (mean values of absorbance after compounds’ incubation/mean values of controls’ absorbance) × 100

Statistical analysis of the biological activities was performed using GraphPad Prism version 5.00 for Windows (GraphPad^®^ Software, San Diego, CA, USA). Statistical significance was determined using a one-way analysis of variance (ANOVA) followed by a Dunnett’s or Bonferroni’s multiple comparison test and was considered statistically significant when differences between experimental groups had *p* values < 0.05. Data are shown as mean ± standard error of mean (SEM). Sigmoid dose-response curves for cell lines were plotted to calculate the concentration, in each incubation period, which kills 50% of cells (LC50): data were fit to a one-site model with a non-linear regression analysis using GraphPad Prism software (version 5; GraphPad Software Inc., La Jolla, CA, USA).

## 4. Conclusions

The short-term effects of the five studied plants (*P. viridis*, *B. caapi*, *P. harmala*, *M. tenuiflora* and Dc Ab) on the central nervous system remain, to the best of our knowledge, largely unknown. In fact, this paper clarified, for the first time, new findings regarding the phytochemical composition of the decoctions of the previously mentioned plants and of the mixture of *P. viridis* and *B. caapi* (traditional ayahuasca preparation) and *P. viridis* and *P. harmala*, along with their in vitro cytotoxicity in dopaminergic neuronal cells, being dose dependent. These results suggest that the synergetic effect of compounds present in each plant exert neurotoxicity. This is significant since one of the target organs affected by the intake of such substances is the brain. Nonetheless, more studies would be important to assess chronic effects of the compounds and the cellular mechanisms responsible for their cytotoxicity, even the determination of these compounds in biological samples.

## Figures and Tables

**Table 1:** Groups of the main tryptamine and harmala alkaloids and phenolic compounds identified in the methanol extracts by UHPLC-Q/TOF-MS.

Compounds	tR	Molecular Formula	Monoisotopic Mass	[M − H]^−^	[M + H]^+^	In-Source Fragments
**Tryptamine Alkaloids**						
bufotenine (5-HO-DMT)	6.4	C_12_H_16_N_2_O	204.1263	--	205.1341	160.0762 134.0604
psilocin (4-HO-DMT)	7.3	C_12_H_16_N_2_O	204.1263	--	205.1341	160.0762 134.0604
*N*,*N*-dimethyltryptamine (DMT)	8.1	C_12_H_16_N_2_	188.1313	--	189.1392	144.0829 118.0648
5-methoxybufotenine (5-MeO-DMT)	8.5	C_13_H_18_N_2_O	218.1419	--	219.1491	174.0912 130.1588
**Harmala Alkaloids**						
tetrahydroharmol	7.3	C_12_H_14_N_2_O	202.1106	--	203.1190	174.0919
harmalol	8.8	C_12_H_12_N_2_O	200.0949	--	201.1028	160.0757
harmol	9.2	C_12_H_10_N_2_O	198.0793	--	199. 0960	--
tetrahydroharmine (THH)	9.4	C_13_H_16_N_2_O	216.1263	--	217.1331	200.0951, 188.1070
harmane	9.7	C_12_H_10_N_2_	182.0844	--	183.0941	--
harmaline (HML)	9.8	C_13_H_14_N_2_O	214.1106	213.1069	215.1167	200.0943, 174.0908
harmine (HMN)	10.4	C_13_H_12_N_2_O	212.0950	211.0910	213.1037	198.0788, 170.0845
**Phenolic Compounds**						
**Hydroxybenzoic Acids**						
gallic acid	3.0	C_7_H_6_O_5_	170.0215	169.0149	--	
protocatechuic acid	6.5	C_7_H_6_O_4_	154.0266	153.0201	--	
4-hydroxybenzoic acid	8.1	C_7_H_6_O_3_	138.0317	137.0271	--	
gentisic acid	8.5	C_7_H_6_O_4_	154.0266	153.0197	--	
salicylic acid	11.5	C_7_H_6_O_3_	138.0317	137.0271	--	
**Hydroxycinnamic Acids**						
3-chlorogenic acid	9.0	C_16_H_18_O_9_	354.0951	353.0803	--	
5-chlorogenic acid	9.7	C_16_H_18_O_9_	354.0951	353.0803	--	
Flavonoids-Flavanols						
(+)-catechin	8.6	C_15_H_14_O_6_	290.079	289.0723	--	
(−)-epicatechin	9.5	C_15_H_14_O_6_	290.079	289.0723	--	
quercetin-3-*O*-galactoside	10.9	C_21_H_20_O_12_	464.0955	463.0888	--	
quercetin-3-*O*-glucoside	11.1	C_21_H_20_O_12_	464.0955	463.0888	--	
quercetin-3-*O*-rutinoside	11.1	C_27_H_30_O_16_	610.1534	609.1461	--	
kaempferol-3-*O*-glucoside	11.6	C_21_H_20_O_11_	448.1006	447.0933	--	
kaempferol-3-*O*-rutinoside	11.5	C_27_H_30_O_15_	594.1615	593.1542	--	
**Flavonoids-Dihydrochalcone**						
phlorizin	11.3	C_21_H_24_O_10_	436.1369	435.1302	--	

tR: retention time (min).
