# Correction: Simão, A.Y., et al. Evaluation of the Cytotoxicity of Ayahuasca Beverages. *Molecules* 2020, *25*, 5594

**DOI:** 10.3390/molecules26051236

**Published:** 2021-02-25

**Authors:** Ana Y. Simão, Joana Gonçalves, Ana Gradillas, Antonia García, José Restolho, Nicolás Fernández, Jesus M. Rodilla, Mário Barroso, Ana Paula Duarte, Ana C. Cristóvão, Eugenia Gallardo

**Affiliations:** 1 Centro de Investigação em Ciências da Saúde (CICS-UBI), Universidade da Beira Interior, Avenida Infante D. Henrique, 6200-506 Covilhã, Portugal; anaaysa95@gmail.com (A.Y.S.); janitagoncalves@hotmail.com (J.G.); jose.restolho@ubi.pt (J.R.); apcd@ubi.pt (A.P.D.); 2 Laboratório de Fármaco-Toxicologia, UBIMedical, Universidade da Beira Interior, Estrada Municipal 506, 6200-284 Covilhã, Portugal; 3 CEMBIO, Center for Metabolomics and Bioanalysis, Facultad de Farmacia, Universidad San Pablo CEU, CEU Universities, Campus Monteprincipe, Boadilla del Monte, 28668 Madrid, Spain; gradini@ceu.es (A.G.); antogar@ceu.es (A.G.); 4 Cátedra de Toxicología y Química Legal, Laboratorio de Asesoramiento Toxicológico Analítico (CENATOXA), Facultad de Farmacia y Bioquímica, Universidad de Buenos Aires, Junín 956, Ciudad Autónoma de Buenos Aires (CABA), Buenos Aires C1113AAD, Argentina; nfernandez@ffyb.uba.ar; 5 Materiais Fibrosos e Tecnologias Ambientais—FibEnTech, Departamento de Química, Universidade da Beira Interior, Rua Marquês D’Ávila e Bolama, 6201-001 Covilhã, Portugal; rodilla@ubi.pt; 6 Instituto Nacional de Medicina Legal e Ciências Forenses, Serviço de Química e Toxicologia Forenses, Delegação do Sul, Rua Manuel Bento de Sousa n.°3, 1169-201 Lisboa, Portugal; mario.j.barroso@inmlcf.mj.pt; 7 NEUROSOV, UBIMedical, Universidade da Beira Interior, Estrada Municipal 506, 6200-284 Covilhã, Portugal

This paper [1] reports important data on the cytotoxicity of Ayahuasca beverages on dopaminergic cells. However, some of the reported information could be misinterpreted; as such, we would like to clarify a few issues in order to avoid misleading the reader.

Indeed, we believe that it is important to state that the plant extracts used in this research work were purchased from a smartshop and did not come from their natural environment. As such, we have no information whether or not those extracts were modified or fortified in order to be more attractive to consumers, which would have impacted the obtained phytochemical composition.

Therefore, the following modifications should be considered, namely in the Results and Discussion, Materials and Methods, and Conclusions sections. In order to be easier for the reader to follow, a short text from the original manuscript is cited together with the respective modification. 

## 2. Results and Discussion

### 2.1. Characterization of Alkaloid Derivatives and Other Compounds

Original: 

Through GC–MS analysis in scan (Figure 1) and SIM modes, it was possible to identify and determine the concentrations of DMT, HMN, THH, and HML. The concentrations found were 2.06 µg/mL of DMT and 0.20 µg/mL of HML for *P. viridis*. In the case of *M. tenuiflora*, the concentration of DMT was inferior (1.70 µg/mL); 0.74 µg/mL of HMN was also found. As for *B. caapi*, *P. harmala*, and Dc Ab, all plant extracts contained THH, HML, and HMN at different concentration levels. The first contained 1.22, 0.99 and 12.81 µg/mL, respectively. As for *P. harmala*, the levels were 7.46, 26.95, and 24.27 µg/mL; it is important to note that this plant extract was the one that presented the highest levels of beta-carboline alkaloids. Finally, Dc Ab contained 1.11, 0.30, and 0.20 µg/mL of THH, HML, and HMN, respectively [21].

This should be replaced with: 

Through GC–MS analysis in scan (Figure 1) and SIM modes, it was possible to identify and determine the concentrations of DMT, HMN, THH, and HML. The concentrations found were 2.06 µg/mL of DMT and 0.20 µg/mL of HML for *P. viridis*. In the case of *M. tenuiflora*, the concentration of DMT was inferior (1.70 µg/mL); 0.74 µg/mL of HMN was also found. As for *B. caapi*, *P. harmala*, and Dc Ab, all plant extracts contained THH, HML, and HMN at different concentration levels. The first contained 1.22, 0.99 and 12.81 µg/mL, respectively. As for P. harmala, the levels were 7.46, 26.95, and 24.27 µg/mL; it is important to note that this plant extract was the one that presented the highest levels of beta-carboline alkaloids. Finally, Dc Ab contained 1.11, 0.30, and 0.20 µg/mL of THH, HML and HMN, respectively [21]. A few considerations may be made regarding these data. Indeed, the presence of beta-carbolines in *P. viridis* and in *M. tenuiflora* was not in accordance with previous publications [11]. This may be justified by the fact that the samples were not obtained from their natural environment, but were purchased from a smartshop. Therefore, one cannot exclude the possibility that these compounds were added to the samples in order to make them stronger or make them more appealing for customers.

In the same section, after Table 1.

Original: 

Results showed that *P. viridis*, *M. hostilis*, and Dc Ab were the ones that contained the highest levels of tryptamine alkaloids, mainly DMT and 5-OH-DMT [25]. On the other hand, *B. caapi* and *P. harmala* contained the highest levels of beta-carboline harmala alkaloids, mainly HML and HMN (Figure 3), as expected [25].

This should be replaced with: 

Results showed that *P. viridis*, *M. hostilis* and Dc Ab were the ones that contained the highest levels of tryptamine alkaloids, mainly DMT and 5-OH-DMT [25]. On the other hand, *B. caapi* and *P. harmala* contained the highest levels of beta-carboline harmala alkaloids, mainly HML and HMN (Figure 3), as expected [25]. The detection of 5-MeO-DMT in the methanol extracts was not common, and may be justified by the fact that the plant samples were not obtained from their natural environment, and may have been fortified with other compounds to enhance their effects.

## 3. Materials and Methods

### 3.1. Sample Preparation

Original:

Samples of *P. viridis*, *B. caapi*, *M. hostilis,* and *P. harmala*, and the commercial mixture Dc Ab were purchased online from the Shayana Shop (https://www.shayanashop.com, Amsterdam, The Netherlands). 

This should be replaced with:

Samples of *P. viridis*, *B. caapi*, *M. hostilis,* and *P. harmala* and the commercial mixture Dc Ab were not obtained from their natural environment, but rather purchased online from the Shayana Shop (https://www.shayanashop.com, Amsterdam, The Netherlands). This means that these samples could have been fortified with other compounds not usually associated with these particular plants.

## 4. Conclusions

Original: 

The short-term effects of the five studied plants (*P. viridis*, *B. caapi*, *P. harmala*, *M. tenuiflora,* and Dc Ab) on the central nervous system remain, to the best of our knowledge, largely unknown. In fact, this paper clarified, for the first time, new findings regarding the phytochemical composition of the decoctions of the previously mentioned plants and of the mixture of *P. viridis* and *B. caapi* (traditional ayahuasca preparation) and *P. viridis* and *P. harmala*, along with their in vitro cytotoxicity in dopaminergic neuronal cells, being dose-dependent. These results suggest that the synergetic effect of compounds present in each plant exert neurotoxicity. This is significant, since one of the target organs affected by the intake of such substances is the brain. Nonetheless, more studies would be important to assess chronic effects of the compounds and the cellular mechanisms responsible for their cytotoxicity and the determination of these compounds in biological samples.

This should be replaced with:

The short-term effects of the five studied plants (*P. viridis*, *B. caapi*, *P. harmala*, *M. tenuiflora,* and Dc Ab) on the central nervous system remain, to the best of our knowledge, largely unknown. In fact, this paper clarified, for the first time, the in vitro cytotoxicity of ayahuasca tea in dopaminergic neuronal cells, which was found to be dose-dependent. These results suggest a synergetic effect of the compounds present in each plant to exert neurotoxicity. This is significant, since one of the target organs affected by the intake of such substances is the brain. Nonetheless, more studies would be important to assess chronic effects of the compounds and the cellular mechanisms responsible for their cytotoxicity and the determination of these compounds in biological samples. 

These changes have no material impact on the aim of our paper. We apologize for any inconvenience to the readers.
